# Anorectal incontinence among a working‐age population: A cross‐sectional survey of prevalence and epidemiology

**DOI:** 10.1111/codi.70392

**Published:** 2026-02-05

**Authors:** Alexandre Balaphas, Emilie Liot, Vaihere Delaune, Jeremy Meyer, Véronique Gogniat, Christian Toso, Guillaume Meurette, Hubert Vuagnat, Frédéric Ris

**Affiliations:** ^1^ Division of Digestive Surgery University Hospitals of Geneva Geneva Switzerland; ^2^ Department of Surgery University of Geneva Genève Switzerland; ^3^ Nurse direction University Hospitals of Geneva Geneva Switzerland; ^4^ Care directorate University Hospitals of Geneva Geneva Switzerland

**Keywords:** anal incontinence, anorectal incontinence, diabetes mellitus, faecal incontinence, hospital staff survey, Jorge–Wexner score, obstetric risk factors, prevalence, proctologic surgery, Rome IV criteria

## Abstract

**Background:**

Anorectal incontinence is a major health issue due to its economic burden and impact on quality of life. Its true prevalence remains under‐estimated and a matter of debate. Our aim was to evaluate the prevalence of anal incontinence among the collaborators of a tertiary hospital.

**Methods:**

An anonymous survey was distributed to all collaborators of a tertiary hospital using a standardized electronic questionnaire, incorporating Jorge–Wexner and LARS scores and items covering different definitions of anorectal incontinence, along with demographic characteristics and specific medical history.

**Results:**

Among 14,270 collaborators, 2535 filled the questionnaire. This sample was comparable to the total hospital staff concerning age, gender and occupation. Anorectal incontinence (defined by a Jorge–Wexner score ≥ 3) was present in 20.9% of participants. However, only 2.3% fulfilled the strict Rome IV criteria for faecal incontinence. The presence of anorectal incontinence was higher in women (16.2% vs. 4.7%, *p* = 0.001), but the Rome IV criteria were comparable. In women, vaginal delivery was not associated with anal incontinence in univariate and multivariate analyses or with Rome IV criteria after adjustment. Diabetes was markedly associated with the presence of Rome IV criteria (OR: 3.3, 95% CI: 1.09–10.08, *p* = 0.035). History of proctological procedure was also substantially associated with anorectal incontinence and Rome IV criteria (OR: 4, 95% CI: 1.86–8.6, *p* < 0.001).

**Conclusion:**

Prevalence of anal incontinence was higher than expected in an active population, and in this medically sensitized working cohort, traditional obstetric factors appeared less strongly associated with anorectal incontinence than anticipated, suggesting a more complex risk profile.

**What does this paper add to the literature?**

This large hospital staff survey reveals that anorectal incontinence affects roughly one in five active, medically sensitized workers, while only a small fraction meet strict Rome IV criteria. It highlights unexpected, non‐obstetric drivers—particularly diabetes and prior proctological surgery—challenging traditional obstetric risk paradigms in a working‐age population.

## INTRODUCTION

The true prevalence of anal incontinence remains a matter of considerable debate, with reported rates varying dramatically from 1.4% to 19.5% across different studies [1]. This wide variation stems from multiple methodological challenges that have consistently plagued epidemiological research in this field.

First, there exists significant terminological confusion between anal incontinence and faecal incontinence, a distinction not always clearly differentiated by authors. Anal incontinence has been defined by the International Continence Society as the involuntary emission of solid stool, liquid stool or gas, inducing a social or hygiene issue, whereas faecal incontinence is restricted to stools [2]. To address this ambiguity, the International Continence Society has recently proposed the unified term ‘anorectal incontinence’, which encompasses both categories and recognizes that diagnosis should be based on symptoms and signs of incontinence (flatus or stools), potentially assisted by para‐clinical examinations [3].

Second, anal incontinence has historically been inadequately reported and often dismissed as merely a symptom rather than recognized as a distinct medical condition deserving of systematic evaluation and treatment. This perspective has contributed to under‐diagnosis and under‐reporting, particularly given the social stigma associated with these symptoms.

Finally, the taboo nature of anorectal incontinence significantly influences response rates and answer veracity in epidemiological studies. The manner in which questionnaires are designed, distributed and the degree of guaranteed anonymity are critical factors that can dramatically impact the reliability of prevalence estimates.

Integrating these methodological challenges, we identified an opportunity to obtain more accurate prevalence estimates by focusing on hospital workers, who are generally more aware of medical issues and may be more willing to provide honest responses to an anonymous medical questionnaire. The primary aim of this study was therefore to assess the prevalence of anorectal incontinence in this distinct population. The primary objective was to estimate the prevalence of anorectal incontinence using a standardized questionnaire, while secondary objectives were to characterize patterns of anorectal incontinence by age and gender, and to explore associations with obstetric history, medical comorbidities, prior perineal surgery and occupational factors in this medically sensitized working population.

## METHOD

### Study design and ethics considerations

This cross‐sectional survey was conducted at Geneva University Hospitals between February and March 2022. The project was notified to the Geneva ethics committee and was considered as falling outside the scope of Swiss legislation regulating research on human subjects, thus waiving the need for formal ethics committee approval. The survey was anonymous, and the IP addresses of participants were not tracked. This work was reported in accordance with the CROSS guidelines [4]. To ensure consistency, the unified term ‘anorectal incontinence’ was used throughout, and the presence of at least one symptom of incontinence was considered as putative of anorectal incontinence.

### Questionnaire development and validation

Using REDCap (Vanderbilt University, Nashville, TN, USA), a 33‐item comprehensive questionnaire, combining different anal/faecal incontinence symptom criteria and validated French translations of the Jorge–Wexner score [5] and the LARS (Low Anterior Resection Syndrome) score [6] was created (Data S1). The questionnaire also collected comprehensive demographic data, relevant surgical and medical history, gynaecological history, and occupational factors and was validated by an expert panel (FR, GM, HV and an independent expert). The LARS score was selected because it captures a broad spectrum of bowel dysfunction symptoms not evaluated in the Jorge–Wexner score and has been widely used in colorectal practice. Although the questionnaire was not formally pretested in a pilot sample, it was constructed from validated instruments and reviewed by an expert panel (FR, GM, HV and an independent expert).

Given the absence of structured diagnostic criteria specifically for anorectal incontinence, Rome IV faecal incontinence ‘criteria for research purposes’ (Rome IV‐FICR) was used as a surrogate gold standard [7]. These criteria define faecal incontinence as ‘recurrent uncontrolled passage of faecal material for the last 6 months, with 2–4 episodes over 4 weeks’. It is acknowledged that these criteria likely lead to an under‐estimation of the true disease prevalence due to the exclusion of gas incontinence.

### Study population and data collection

An online questionnaire link was distributed to the professional email addresses of all Geneva University Hospital collaborators (*n* = 14,270). This approach constituted a census‐style invitation to the entire hospital workforce, without additional sampling, thereby ensuring a degree of anonymity and the obtention of a large convenience sample. A reminder was sent 1 month later to maximize response rates. A response rate of at least 10% was targeted to achieve a sufficiently large sample size, as the reported prevalence of anorectal incontinence varies widely in the published literature.

To assess the representability of the retrieved data, official statistics of the human resources department were used as a comparison. Data were extracted from RedCap and analyzed using Stata 15 (StataCorp, College Station, TX, USA). AB and JM independently verified and cleaned the data, with ambiguous responses (such as impossible BMI values) treated as missing data. Duplicate or incomplete questionnaires missing incontinence items were excluded from analysis.

### Statistical analysis

Descriptive statistics of variables and graphical representations were performed to assess data distributions. According to this first step, mean with standard deviation or median with interquartile range were used when appropriate. Two‐sided statistical tests were used (Chi square or Fisher's exact test). A *p*‐value <0.05 was considered statistically significant. The complex and multiple dimensions of anorectal incontinence were evaluated by repeating analysis for different items and definitions (Table S1). In this context, anorectal incontinence was primarily operationalized using three complementary outcomes: (i) a Jorge–Wexner score ≥3 as a pragmatic threshold for clinically relevant anorectal symptoms; (ii) the presence of soiling (patients were asked for staining and/or oozing); and (iii) fulfilment of the Rome IV faecal incontinence criteria for research purposes (Rome IV‐FICR). These definitions were chosen a priori based on clinical relevance and existing literature [8]. For the main comparative analyses, these items were selected as primary outcomes, while additional analyses using alternative categorizations were considered exploratory sensitivity analyses intended to assess the robustness of the findings.

For logistic regression models, homoscedasticity and normal distribution of continuous variables were verified, and variables were categorized when necessary. Model goodness‐of‐fit was assessed using the Hosmer–Lemeshow test. Analyses were conducted on a complete‐case basis for most variables, with missing data left un‐imputed and the number of observations reported for each model, with multivariate models adjusting for relevant demographic and clinical confounders.

## RESULTS

### Study population characteristics

**FIGURE 1:**
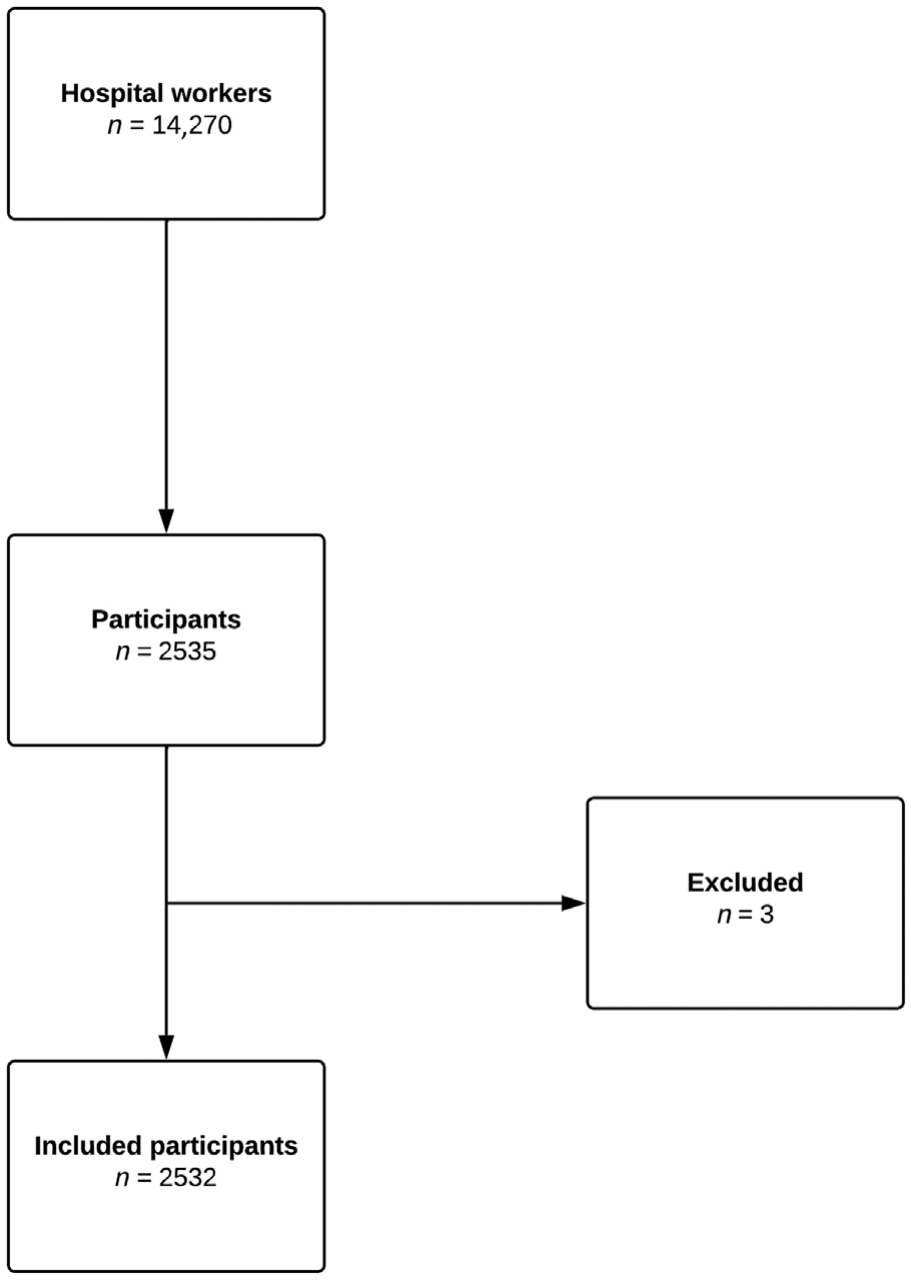
Chart flow of included questionnaires.

The questionnaire was distributed to 14,270 hospital collaborators with active employment contracts during the study period. After two distribution rounds, 2535 responses (17.8% response rate) were collected. Following data review, three questionnaires were excluded due to duplication or incompleteness, yielding a final analytical sample of 2532 participants (Figure 1). The demographic composition included 71.7% women, with 66.7% of women having uni‐ or multiparous history. Occupational distribution reflected typical hospital staffing: 41% nursing staff, 15.4% administration, 13.4% medical staff, 7.7% medical technical staff, 7% therapy staff, 6.2% unclassified positions, 4.8% technical staff, 1.9% logistics, 1.8% cleaning and kitchen staff and 0.8% social services staff. Complete demographic characteristics are summarized in Tables S2 and S3. Importantly, comparison with official hospital statistics based on 12,791 permanent employment contracts confirmed that our sample was similar regarding gender, age distribution and work categories, supporting the external validity of our findings. Medical and gynaecological history of participants are reported in Tables S4 and S5, respectively.

### Prevalence of symptoms of anorectal incontinence

**FIGURE 2:**
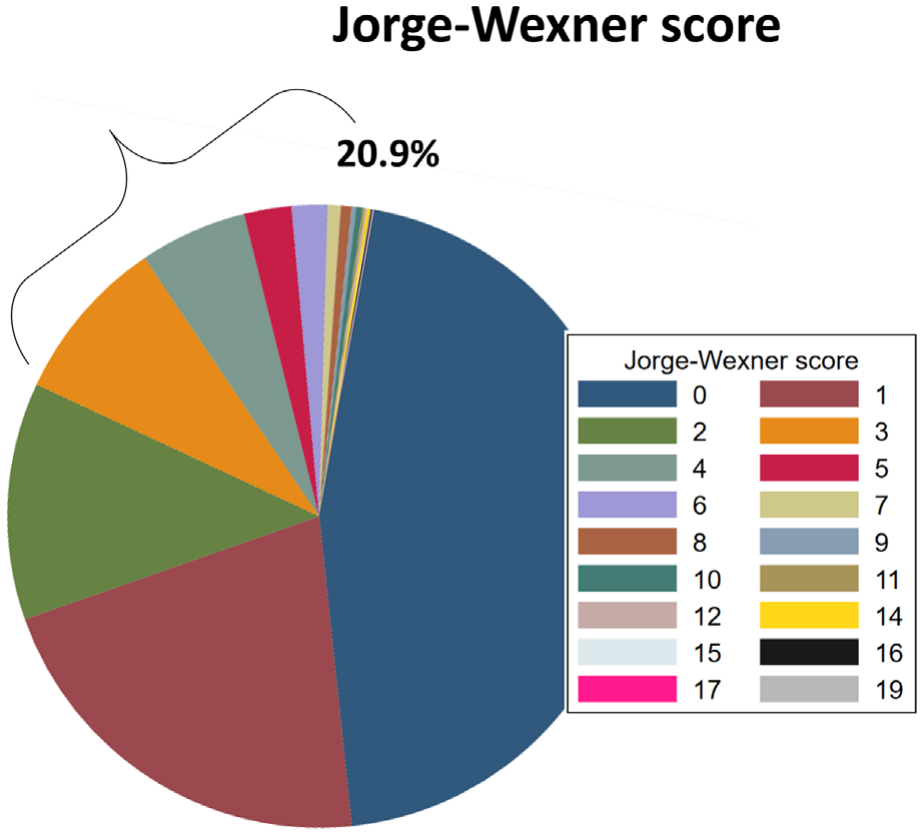
Repartition of Jorge–Wexner score among participants (minimum 0, maximum 20).

The presence of faecal incontinence symptoms was determined according to the Rome IV‐FICR criteria and identified in 2.3% (59/2521) of participants. To account for both faecal and gas incontinence, anal incontinence was further assessed using the Jorge–Wexner scoring system. A threshold score of ≥3 was applied, corresponding to 20.9% of participants (Figure 2) [8]. This threshold was further investigated with an ad‐hoc complementary analysis. Using Rome IV‐FICR criteria as the gold standard for anorectal incontinence, ROC analysis of the Jorge–Wexner score yielded an area under the curve (AUC) of 0.96, indicating excellent discriminative ability. With a cut‐off point of 3, the Jorge–Wexner score achieved sensitivity of 94.8% and specificity of 80.9% for detecting anorectal incontinence (Figure S1). Additional scoring parameters and individual item distributions are summarized in Table 1.

**TABLE 1:** Selected items of the questionnaire. Right column *n*: number of complete cases analysed per variable.

		*n*
Jorge–Wexner score, mean SD	1.4 (2)	2525
LARS median, mean SD	11.4 (9)	2182
Anal incontinence, even rarely, *n* (%)	1375 (54.4)	2528
Anal incontinence, even occasionally, *n* (%)	734 (29)	2528
Faecal incontinence, even rarely, *n* (%)	378 (15)	2528
Faecal incontinence, even occasionally, *n* (%)	145 (5.7)	2528
Faecal urgency		
Never, *n* (%)	1444 (57.1)	2529
< 1×/week, *n* (%)	919 (36.3)	
1×/week or more, *n* (%)	166 (6.6)	
Soiling *n* (%)	191 (7.6)	2520
Reporting faecal incontinence according to Rome IV, *n* (%)	104 (4.1)	2521
Rome IV faecal incontinence, criteria for research, *n* (%)	59 (2.3)	2521

### Gender and age‐related patterns

Significant gender differences emerged in anorectal incontinence prevalence patterns. Women demonstrated higher mean Jorge–Wexner scores compared to men (1.5 ± 0.5 vs. 1.1 ± 0.7, *p* < 0.001) (Table S6). This difference was primarily driven by two specific components: gas incontinence and quality‐of‐life impact, both significantly worse in women. Interestingly, when Rome IV‐FICR criteria were applied, no gender differences were observed. Faecal urgency prevalence was also statistically significantly higher in women compared to men (Table S6). Conversely, soiling prevalence was significantly higher in men than women (10.3% vs. 6.5%, *p* = 0.001) (Table S6).

Age‐related increases in incontinence were observed across both genders, with statistically significant trends toward higher Jorge–Wexner scores in older age categories (Table S7). Among women, two distinct prevalence peaks were identified: the first between ages 35–44 years and the second between ages 55–59 years. Gender differences in incontinence prevalence were most pronounced in younger age groups (25–49 years) but diminished in older populations (Table S7, Figure S2).

### Anorectal incontinence and medical history

Diabetes emerged as an important correlate of anorectal incontinence. In univariate analysis, diabetes was associated with Rome IV‐FICR (OR: 4.3, 95% CI: 1.5–12.4, *p* = 0.007), and this association remained robust after adjustment for gender, age category and BMI (OR: 3.3, 95% CI: 1.09–10.08, *p* = 0.035) (Table S8). However, diabetes duration was not significantly associated with anal incontinence (data not shown).

History of proctological procedures demonstrated particularly strong associations with anorectal incontinence across all measured parameters (Table 2). Specific procedures showed varying patterns of association: haemorrhoid surgery with Jorge–Wexner score ≥3 (adjusted OR: 1.9, 95% CI: 1.03–3.39, *p* = 0.041); fistula surgery with Jorge–Wexner score ≥3 (adjusted OR: 4.58, 95% CI: 1.77–11.87, *p* = 0.002); perianal abscess surgery with soiling (adjusted OR: 7.9, 95% CI: 1.82–34.1, *p* = 0.006); and most notably, anal fissure surgery with Rome IV‐FICR (adjusted OR: 13.4, 95% CI: 4.05–44.49, *p* = 0.001).

**TABLE 2:** Univariate and multivariate analysis of association between anal incontinence items and perineal surgery.

	Univariate logistic regression			Multivariate logistic regression			n
	OR	95% CI	*p* value	OR	95% CI	*p* value	
History of perineal surgery							
Soiling	4.7	2.9–7.6	**<0.001**	4.4	2.7–7.2	**<0.001**	2517
Rome IV faecal incontinence criteria for research	4.9	2.3–10.2	**<0.001**	4	1.9–8.6	**<0.001**	2517
Jorge–Wexner ≥3	2.8	1.9–4.3	**<0.001**	2.4	1.5–3.6	<0.001^a^	2511
History of hemorrhoid surgery							
Soiling	2.7	1.29–5.62	**0.008**	2.4	1.12–5.04	**0.023**	2519
Rome IV faecal incontinence criteria for research	2.7	0.82–8.91	0.104	2	0.6–6.79	0.257	2520
Wexner ≥3	2.3	1.29–4.09	**0.005**	1.9	1.03–3.39	**0.041**	2524
History of fistula surgery							
Soiling	16	6.25–41.11	**<0.001**	14	5.36–36.61	**<0.001**	2519
Rome IV faecal incontinence for research	2.5	0.33–18.95	0.381	2.2	0.29–17.6	0.447	2520
Jorge–Wexner ≥3	4.8	1.89–12.25	**0.001**	4.58	1.77–11.87	**0.002**	2524
History of para‐anal abscess surgery							
Soiling	7.4	1.76–31.28	**0.006**	7.9	1.82–34.1	**0.006**	2519
Rome IV faecal incontinence for research	6	0.73–49.95	0.095	8.5	0.95–77.11	0.056	2520
Jorge–Wexner ≥3	2.3	0.54–9.58	0.260	2.9	0.68–12.37	0.150^a^	2524
History of anal fissure surgery							
Soiling	12.8	5–32.51	**<0.001**	13.88	5.29–36.15	**<0.001**	2519
Rome IV faecal incontinence for research	12.72	4.06–39.88	**<0.001**	13.4	4.05–44.49	**<0.001**	2520
Jorge–Wexner ≥3	4.8	1.89–12.25	**0.001**	4.2	1.61–10.72	**0.003** ^a^	2524
History of organ prolapse surgery							
Soiling	3.5	1.15–10.85	**0.027**	3.5	1.13–11.04	**0.030**	2519
Rome IV faecal incontinence for research	5.4	1.21–23.88	**0.027**	3.9	0.85–17.95	0.080^a^	2520
Jorge–Wexner ≥3	3	1.20–7.80	**0.019**	2.2	0.82–5.47	0.121^a^	2524

*Note*: Bold indicate *p* value <0.05. Highlighted in green are rows where association was confirmed with multivariate analysis.

Abbreviations: CI, confidence interval; OR, odds ratio.

^a^ Model not valid.

Regarding obesity, a significant association between BMI category and anorectal incontinence was identified (Table S9). Participants with BMI 35–40 (Grade 2 obesity) demonstrated increased odds for anorectal incontinence compared to those with normal BMI (OR: 2.15, 95% CI: 1.17–3.93, *p* = 0.013). All obesity categories (BMI ≥ 30) were significantly associated with soiling symptoms, with odds ratios ranging from 2.02 to 4.45.

### Obstetrical or gynaecological history and anorectal incontinence

In univariate analyses, pregnancy and vaginal birth or delivery by caesarean section were weakly associated with anorectal incontinence. These associations were no longer statistically significant when adjusted for age category (Table 3). Similarly, compared to nulliparous participants, women with multiple childbirths showed slight increases in anorectal incontinence measures, but these associations disappeared after adjustment for age category, BMI and diabetes status (Table S10). The one notable exception was history of perineal laceration during vaginal delivery, which maintained significant associations with anorectal incontinence measures even after comprehensive adjustment, though with relatively modest effect sizes (OR ranging from 1.3 to 2.38) (Table 3). Preventative episiotomy was not associated with a reduced prevalence of anorectal incontinence (Table S11). Finally, history of hysterectomy was not associated with anorectal incontinence (Table 3).

**TABLE 3:** Association between gynaecological and obstetrical history and anal incontinence panel items.

	Univariate logistic regression			Multivariate logistic regression			*n*
	OR	95% CI	*p* value	OR	95% CI	*p* value	
History of delivery							
Soiling	1.3	0.83–1.89	0.284	1.1	0.65–1.65	0.880	1800
Rome IV faecal incontinence for research	2	0.97–4.24	0.060	1.9	0.84–4.4	0.120^a^	1757
Wexner ≥3	1.7	1.33–2.19	**<0.001**	1.3	0.95–1.7	0.112^a^	1797
History of perineal laceration during delivery							
Soiling	2.38	1.52–3.73	**<0.001**	2.48	1.57–3.93	**<0.001**	1193
Rome IV faecal incontinence for research	2.15	1.1–4.18	**0.025**	2.37	1.2–4.69	**0.013**	1186
Wexner ≥3	1.3	1–1.7	0.05	1.4	1.04–1.79	**0.025**	1191
History of hysterectomy							
Soiling	1.08	0.63–1.84	0.790	1.19	0.69–2.06	0.540	1800
Rome IV faecal incontinence for research	0.67	0.37–1.23	0.199	0.81	0.43–1.5	0.499	1801
Wexner ≥3	0.9	0.68–1.2	0.486	1.1	0.81–1.47	0.559	1797

*Note*: History of delivery: Adjustment for age category. Delivery lacerations: Adjustment for BMI, diabetes and age category. Hysterectomy: Adjustment for age. Bold highlighted *p* values under 0.05. Highlighted in green are rows where association was confirmed with multivariate analysis.

^a^ Model not valid.

### Occupational and lifestyle factors and anorectal incontinence

Several unexpected occupational associations emerged from our analysis. Participants working night shifts demonstrated lower rates of anorectal incontinence, though after age adjustment, only the Jorge–Wexner score ≥3 item remained significant (adjusted OR: 0.79, 95% CI: 0.64–0.99, *p* = 0.038) (Table S12).

Compared to nursing staff, technical workers and cleaning/kitchen staff showed increased associations with anorectal incontinence after gender adjustment. Technical staff demonstrated particularly elevated risk for Rome IV‐FICR (OR: 3.42, 95% CI: 1.2–9.76, *p* = 0.022), while cleaning and kitchen staff showed similar increases (OR: 2.41, 95% CI: 1.3–4.45, *p* = 0.005) (Table S13).

## DISCUSSION AND CONCLUSIONS

This study provides novel insights into anorectal incontinence epidemiology through systematic assessment of a large, medically informed population. Our finding that 20.9% of working‐age adults experience anorectal symptoms (Jorge–Wexner score ≥3) substantially exceeds most literature estimates and likely reflects the combined advantages of improved detection sensitivity and reduced reporting bias inherent in our hospital worker population.

The definition of Jorge–Wexner score ≥3, also utilized by Postillon et al. [8] in similar research, provides a threshold that captures patients with at least mild but recurring symptoms of both faecal and gas incontinence. Moreover, close alignment between the study population and hospital workers demographics strengthens internal validity with respect to this medically oriented working population, while the large sample size (>2500 participants) provides adequate power for subgroup analyses. The anonymous nature of data collection likely enhanced reporting accuracy for this sensitive topic.

### Gender effects are modest and age‐dependent

We demonstrated that gender differences were restricted to some aspects of anorectal incontinence (Table S6). This finding is coherent with the literature, where gender differences in anorectal incontinence prevalence are subtle and inexistent when isolated faecal incontinence is considered [9, 10]. However, more faecal urgencies were recorded in women than in men, a finding completely different from the study by Santacruz et al. [9] where no statistically significant differences were found between genders for this symptom. The increased prevalence in women of urge‐type anorectal incontinence has been previously described by others and seems to be related to child birth [11, 12]. It could be explained by traumatism to pelvic nerves during vaginal delivery [11, 13]. Interestingly, soiling was more prevalent in men compared to women. This may be due to the higher prevalence of haemorrhoidal disease in men compared to women [14].

Increasing age, a well‐recognized factor of anal incontinence, was associated with anorectal incontinence in this survey (Table S7). Factors such as sequelae of cerebrovascular events or deterioration of mobility could be implicated in the increased prevalence of anorectal incontinence in the elderly [15]. Gender differences were also observed in anorectal incontinence prevalence, but restricted only to some age categories such as young women (Table S7). Notably, we observed two peaks: the first one between ages 35 and 44 years and the second one between ages 55 and 59 years. The first peak could be related to the acute effects of childbirth, and the second one to a chronic effect after childbirth and/or the delayed effect of menopause which is a known factor of anorectal incontinence [16]. In men, we observed a late peak between ages 55 and 59 years but there were no statistically significant gender differences for older age categories. All these findings confirm other studies reporting an increased or similar prevalence of anorectal incontinence in older men compared to older women [15, 17]. This could be linked to the increased incidence of prostate adenocarcinoma in older men, some of them treated by radiotherapy [17, 18]. However, these potential contributors were not specifically captured in our questionnaire and should therefore be regarded as speculative.

### Challenging conventional obstetric risk paradigms

In the investigated population, standard obstetric factors showed weaker associations with anorectal incontinence than might be expected from traditional paradigms. After adjustment for key confounders, most obstetric variables lost statistical significance, with the notable exception of perineal laceration during delivery, which retained modest associations. Indeed, recent studies confirm that the main obstetrical risk factor for anorectal incontinence is a complicated delivery [16, 19, 20].

These observations suggest that, within this specific cohort, the contribution of obstetric factors to anorectal incontinence may be less dominant than in other settings and should be interpreted as hypothesis‐generating rather than definitive. Additionally, a protective effect of caesarean section was not observed (Table S14) [21]. This is probably due to the fact that details on the chronology and indications for caesarean section were not collected.

### Medical comorbidities and perineal surgery history as primary risk factors

While the association between BMI and urinary incontinence is well demonstrated [22], the role of obesity in anorectal incontinence is still debated as larger cohorts, like the Nurses' Health Study including 51,708 women, did not find any association between faecal incontinence and BMI [23]. However, the report from the Nurses' Health Study was restricted to women with stool incontinence that was evaluated with a single question about ‘accidental bowel leakage’. In our cohort, we found an association between anorectal incontinence and grade 2 obesity (WHO classification) (Table S9). Participants with a BMI category >40 had no statistically significant association with anorectal incontinence regarding these items, probably as the result of a lack of power in this category representing <1% of the total cohort. However, obesity of all types (BMI ≥ 30) was statistically significantly associated with soiling, with an OR ranging from 2.02 to 4.45 when compared with a normal BMI (Table S9). Association between high BMI and anorectal incontinence was also demonstrated by Postillon et al. [8] Excess weight induces a chronic increase in abdominal pressure as confirmed by physiological studies on urinary incontinence in obese patients where the causality has been demonstrated [22, 24]. Thus, it is likely that obesity participates in the pathophysiology of anorectal incontinence.

In our cohort, only a small proportion of collaborators (1.8%) reported diabetes. Detailed information such as diabetes type was not asked in the questionnaire for data protection purposes. An association between diabetes and several anorectal incontinence outcomes was observed, although the limited number of diabetic participants restricts the precision of these estimates and warrants cautious interpretation. Diabetes can alter anorectal continence through diabetic neuropathy or direct internal sphincter muscle alterations [25, 26, 27]. This was already reported in the literature [28] with, however, lower OR [29].

The association between perineal surgery and anorectal incontinence was consistent across multiple outcomes, with elevated odds ratios observed for several procedures and a statistically significant association for almost all items of the anorectal incontinence panel. Differences among the different items of the panel highlighted that anorectal incontinence could occur through different mechanisms. Lesions of the IAS, that can occur as a complication of haemorrhoid surgery, induce passive faecal incontinence characterized by spontaneous and involuntary passage of solid stools [30]. Passive faecal incontinence is characterized by low resting anal pressure on anorectal manometry [13]. Fissure surgery, and especially fistula surgery, can have an impact on both the IAS and external anal sphincter (EAS), characterized by passive incontinence and also an inability to repress the defecation reflex [13]. However, it should be recognized that a single perineal pathology can be managed using several distinct approaches, each carrying different risks of anorectal incontinence. Such detailed technical aspects of surgical procedures were not explored in this study.

Finally, collaborators doing night shift seem to have less anorectal incontinence but may be younger. Using a logistic regression model, we were able to adjust on age category and observed a fading of this association. Another explanation is the reduced workload during the night or at least its division, with the possibility to lie down for some categories of workers. Indeed, lifting weights as a sport or occupation has been linked in women with urinary incontinence, and even anorectal incontinence in several studies [31, 32, 33, 34]. We were therefore not surprised to observe higher odds of anorectal incontinence in technical and cleaning/kitchen staff compared to nurses. However, these findings should be considered exploratory, as detailed occupational exposures and workplace constraints (e.g. access to toilets, workload, ergonomic factors) were not collected. (Table S13).

### Limitations and future directions

Several limitations deserve acknowledgment. First, the response rate of 17.8%, although yielding a large absolute sample, raises the possibility of responder bias; individuals with symptoms may have been either more or less likely to participate than asymptomatic colleagues, and data on non‐responders were not available. Second, the study population consisted exclusively of hospital employees, which may reflect a higher level of health awareness (‘medical sensitization’) but limits the generalizability of the findings to the broader community. Furthermore, retired individuals were excluded from the study, which also restricts the applicability of the results to the elderly.

Third, our design precludes any inference about temporality or causality between risk factors and anorectal incontinence. Fourth, despite the use of validated instruments, the entire questionnaire was not pretested and certain constructs (e.g. anorectal incontinence defined by a Jorge–Wexner threshold) represent pragmatic operational definitions rather than established diagnostic criteria.

Fifth, the granularity of some variables was limited: information on diabetes type and duration, surgical techniques, and the chronology and indications for obstetric and gynaecological interventions was not available, and small exposed subgroups (e.g., diabetic participants or specific obstetric strata) restricted statistical power. Finally, important workplace‐related confounders such as physical workload, ergonomic constraints, occupational stress and access to toilets were not captured.

These limitations underline that the associations reported in this survey should be viewed as exploratory and primarily hypothesis‐generating.

## CONCLUSION

This study reveals that anorectal incontinence affects approximately one in five working adults when systematically assessed using validated instruments, representing a substantially higher prevalence than previously recognized. The association analysis challenges traditional risk stratification models by demonstrating that diabetes and prior anorectal surgery might be more predictive of incontinence than historically emphasized obstetric factors.

Future research should focus on longitudinal studies to establish causal relationships and develop evidence‐based prevention strategies for this under‐recognized condition that significantly impacts quality of life.

## AUTHOR CONTRIBUTIONS

**Vaihere Delaune:** Supervision; writing – original draft; writing – review and editing. **Christian Toso:** Supervision; resources; investigation; conceptualization; writing – review and editing. **Jeremy Meyer:** Conceptualization; methodology; supervision; writing – original draft. **Frédéric Ris:** Conceptualization; supervision; writing – review and editing. **Guillaume Meurette:** Validation; writing – review and editing. **Emilie Liot:** Validation; supervision; writing – review and editing. **Alexandre Balaphas:** Conceptualization; investigation; writing – original draft; methodology; writing – review and editing; formal analysis; data curation. **Véronique Gogniat:** Conceptualization; formal analysis; project administration. **Hubert Vuagnat:** Conceptualization; methodology; investigation; validation; supervision; writing – original draft; writing – review and editing.

## FUNDING INFORMATION

The authors have nothing to report.

## CONFLICT OF INTEREST STATEMENT

The authors disclose no conflict of interest.

## ETHICS STATEMENT

This study was conducted using fully anonymized survey data. The local ethics review board waived the requirement for formal ethics committee approval. Data protection and confidentiality were ensured in accordance with applicable regulations, the recommendations of the International Committee of Medical Journal Editors (ICMJE), and the principles of the Declaration of Helsinki.

## Supporting information


Figure S1.

Figure S2.

Table S1.

Table S2.

Table S3.

Table S4.

Table S5.

Table S6.

Table S7.

Table S8.

Table S9.

Table S10.

Table S11.

Table S12.

Table S13.

Table S14.

Data S1.

## Data Availability

The data that support the findings of this study are available from the corresponding author upon reasonable request. Data can be shared on demand.
