# Supplementary material for: Anorectal incontinence among a working‐age population: A cross‐sectional survey of prevalence and epidemiology
Source: Colorectal Dis. 2026 Feb 5;28(2):e70392. doi: 10.1111/codi.70392 (PMC12876054; doi:10.1111/codi.70392)
Supplement: Supplementary file 1 — Figure S1. [file CODI-28-0-s016.docx]

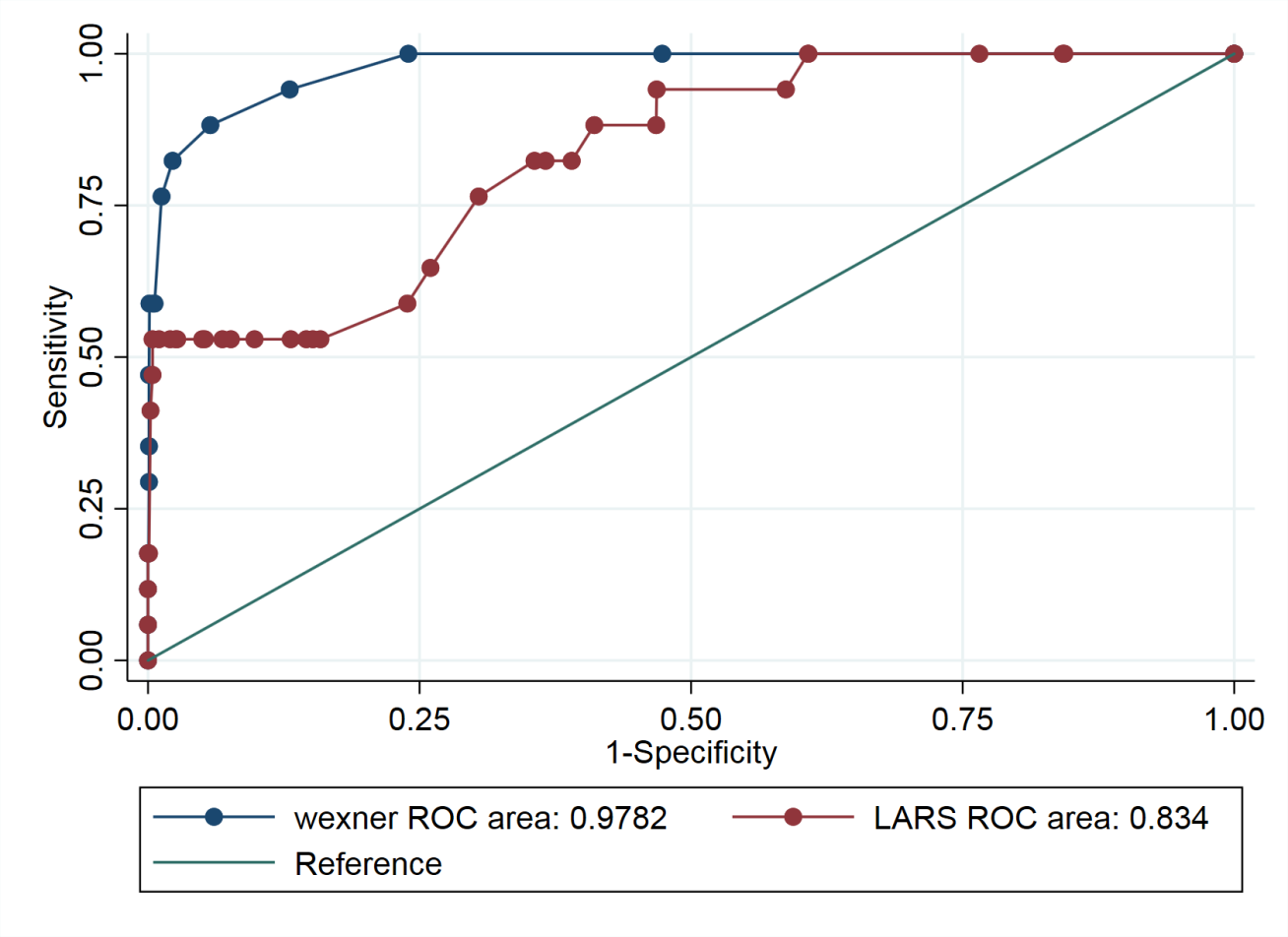


| Cutoff Jorge-Wexner score | Sensitivity (%) | Specificity (%) |
| --- | --- | --- |
| ≥ 1 | 100 | 46.5 |
| ≥ 2 | 98.3 | 68.3 |
| ≥ 3 | 94.8 | 80.9 |
| ≥ 4 | 86.2 | 89.4 |
| ≥ 5 | 81 | 95 |
| ≥ 6 | 65.5 | 97.2 |
| ≥ 7 | 46.6 | 98.7 |
| ≥ 8 | 39.7 | 99.2 |
| ≥ 9 | 34.5 | 99.6 |
| ≥ 10 | 25.9 | 99.7 |
| ≥ 11 | 18.9 | 99.8 |
| ≥ 12 | 13.8 | 99.8 |
| ≥ 14 | 10.3 | 99.9 |
| ≥ 15 | 5.2 | 100 |
| ≥ 16 | 5.2 | 100 |
| ≥ 17 | 3.5 | 100 |
| ≥ 19 | 1.7 | 100 |

**Figure S1** Receiver operating characteristic (ROC) curves of Jorge-Wexner and LARS scores using the Rome IV criteria for research as a gold standard. The accompanying chart details the sensitivity and specificity according to different cutoff of Jorge-Wexner score.
