# Supplementary material for: Anorectal incontinence among a working‐age population: A cross‐sectional survey of prevalence and epidemiology
Source: Colorectal Dis. 2026 Feb 5;28(2):e70392. doi: 10.1111/codi.70392 (PMC12876054; doi:10.1111/codi.70392)
Supplement: Supplementary file 2 — Figure S2. [file CODI-28-0-s008.docx]

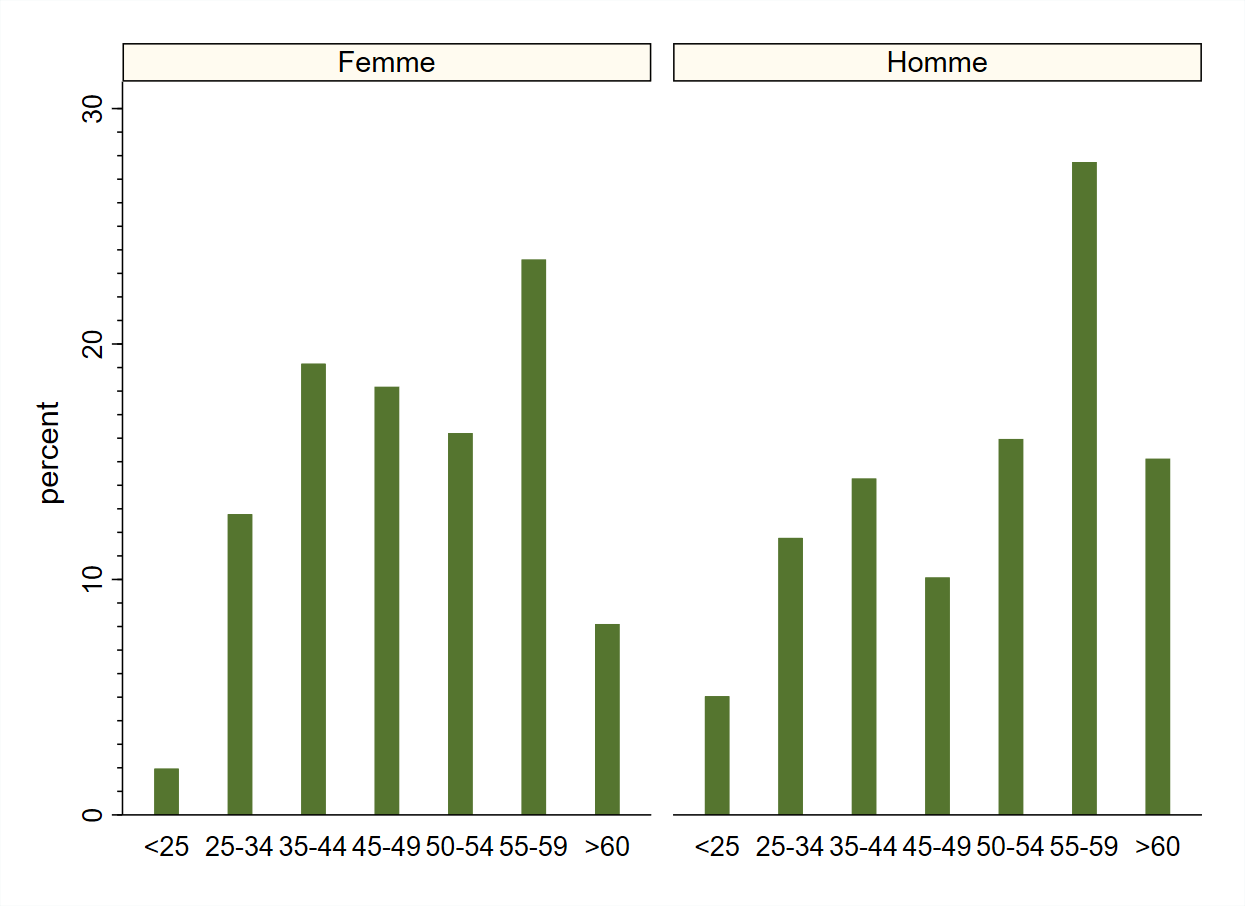


Men

Women

Figure S2 Repartition of participants meeting a Jorge-Wexner of score of 3 or above by gender and age categories.
