# Supplementary material for: Anorectal incontinence among a working‐age population: A cross‐sectional survey of prevalence and epidemiology
Source: Colorectal Dis. 2026 Feb 5;28(2):e70392. doi: 10.1111/codi.70392 (PMC12876054; doi:10.1111/codi.70392)
Supplement: Supplementary file 3 — Table S1. [file CODI-28-0-s010.docx]

|  | Item | Type of incontinence |
| --- | --- | --- |
| A | Anal incontinence, even rarely | Gas and stools |
| B | Anal incontinence, even occasionally | Gas and stools |
| C | Fecal incontinence, even rarely | Stools |
| D | Fecal incontinence, even occasionally | Stools |
| E | Soiling | Stools |
| F | Reporting fecal incontinence according to Rome | Stools |
| G | Rome IV fecal incontinence | Stools |
| H | Jorge-Wexner ≥ 3 | Gas and stools |

**S1** Types of items and their associated symptoms
