# Supplementary material for: Anorectal incontinence among a working‐age population: A cross‐sectional survey of prevalence and epidemiology
Source: Colorectal Dis. 2026 Feb 5;28(2):e70392. doi: 10.1111/codi.70392 (PMC12876054; doi:10.1111/codi.70392)
Supplement: Supplementary file 4 — Table S2. [file CODI-28-0-s012.docx]

|  |  | **Participants** | | **Hospital staff** | |
| --- | --- | --- | --- | --- | --- |
|  | **n** | 2’531 | | 12’791 | |
|  |  | **Women** | **Men** | **Women** | **Men** |
|  | **Gender, n (%)** | 1815 (71.7) | 716 (28.3) | 8790 (68.7) | 4001 (31.3) |
| **Age category, n (%)** | **<25 yo** | 45 (1.9) | 22 (0.9) | 66 (0.5) | 28 (0.1) |
|  | **25-34 yo** | 407 (16.1) | 161 (6.4) | 2179 (17) | 878 (6.9) |
|  | **35-44 yo** | 427 (16.9) | 174 (6.9) | 2249 (17.6) | 1067 (8.3) |
|  | **45-49 yo** | 246 (9.6) | 97 (3.8) | 1160 (9.1) | 560 (4.4) |
|  | **50-54 yo** | 264 (10.4) | 101 (4) | 1168 (9.1) | 512 (4) |
|  | **55-59 yo** | 304 (12) | 116 (4.6) | 1265 (9.9) | 577 (4.5) |
|  | **>60 yo** | 122 (4.8) | 45 (1.8) | 703 (5.5) | 389 (3) |
| **Work Category, n (%)** | **Nursing staff** | 842 (33.3) | 195 (7.7) | 4675 (36.5) | 1110 (8.7) |
|  | **Administration staff** | 308 (12.2) | 83 (3.3) | 1289 (10.1) | 511 (4) |
|  | **Medical staff** | 185 (7.3) | 155 (6.1) | 1100 (8.6) | 963 (7.5) |
|  | **Medical technical staff** | 131 (5.2) | 63 (2.5) | 561 (4.4) | 321 (2.5) |
|  | **Therapy staff** | 158 (6.2) | 18 (0.7) | 436 (3.4) | 102 (0.8) |
|  | **Technical staff** | 15 (0.6) | 106 (4.2) | 29 (0.2) | 306 (2.4) |
|  | **Logistic staff** | 24 (1) | 25 (1) | 71 (0.6) | 187 (1.5) |
|  | **Cleaning and kitchen staff** | 24 (1) | 21 (0.8) | 494 (3.9) | 490 (3.8) |
|  | **Social staff** | 19 (0.8) | 1 (<1) | 148 (1.2) | 14 (0.1) |
|  | **Other** | 110 (6.1) | 49 (6.9) | NA | NA |

**Table S2** Comparison between sampled collaborators and official statistics of the hospital regarding gender, age and occupation.
