# Supplementary material for: Anorectal incontinence among a working‐age population: A cross‐sectional survey of prevalence and epidemiology
Source: Colorectal Dis. 2026 Feb 5;28(2):e70392. doi: 10.1111/codi.70392 (PMC12876054; doi:10.1111/codi.70392)
Supplement: Supplementary file 5 — Table S3. [file CODI-28-0-s015.docx]

|  |  | **n** |
| --- | --- | --- |
| **Female (%)** | 1816 (71.7) | 2532 |
| **Male n (%)** | 716 (28.3) | 2532 |
| **Age cat n (%) [years old]** |  | 2531 |
| <25 | 67 (2.7) |  |
| 25-34 | 568 (22-4) |  |
| 35-45 | 601 (23.8) |  |
| 45-49 | 343 (13.6) |  |
| 50-54 | 365 (14.5) |  |
| 55-59 | 420 (16.6) |  |
| >60 | 167 (6.6) |  |
| **BMI mean ± SD [kg/m2]** | 24.6 ± 4.5 | 2503 |
| **BMI n (%) [kg/m2]** |  |  |
| <18.5 | 68 (2.7) |  |
| 18.5-24.9 | 1493 (59.7) |  |
| 25-30 | 671 (26.8) |  |
| 30-35 | 201 (8) |  |
| 35-40 | 49 (2) |  |
| ≥40 | 21 (0.8) |  |
| **Smoking n (%)** | 466 (18.5) | 2520 |
| **Night shift n (%)** | 926 (36.8) | 2520 |

**Table S3** Demographic characteristics and habits of participants. SD : standard deviation
