# Supplementary material for: Anorectal incontinence among a working‐age population: A cross‐sectional survey of prevalence and epidemiology
Source: Colorectal Dis. 2026 Feb 5;28(2):e70392. doi: 10.1111/codi.70392 (PMC12876054; doi:10.1111/codi.70392)
Supplement: Supplementary file 6 — Table S4. [file CODI-28-0-s014.docx]

|  |  | n |
| --- | --- | --- |
| Diabetes n (%) | 45 (1.8) | 2521 |
| Duration of diabetes md (range) [years] | 4 (0-57) | 44 |
| Perineal trauma* (female) | 132 (7.3) | 1801 |
| Perineal trauma (male) | 94 (13.2) | 712 |
| History of proctological surgery n (%) | 97 (3.9) | 2519 |
| Hemorrhoid surgery n (%) | 51 (2) | 2532 |
| Fistula surgery n (%) | 18 (0.7) | 2532 |
| Abscess surgery | 8 (0.3) | 2532 |
| Anal fissure surgery | 18 (0.7) | 2532 |

**Table S4** Medical history of participants. Md:median. n: number of complete case analyzed per variable. Right column n: number of complete case analyzed per variable *Perineal trauma in women other than obstetrical
