# Supplementary material for: Anorectal incontinence among a working‐age population: A cross‐sectional survey of prevalence and epidemiology
Source: Colorectal Dis. 2026 Feb 5;28(2):e70392. doi: 10.1111/codi.70392 (PMC12876054; doi:10.1111/codi.70392)
Supplement: Supplementary file 7 — Table S5. [file CODI-28-0-s017.docx]

|  |  | n |
| --- | --- | --- |
| **Female n (%)** | 1816 (71.7) | 2532 |
| **Nulliparous n (%)** | 600 (33.3) | 1800 |
| **Primiparous n (%)** | 295 (16.4) | 1800 |
| **Multiparous n (%)** | 905 (50.3) | 1800 |
| **Number of vaginal delivery** |  | 1198 |
| 0 n (%) | 180 (15) |  |
| 1 n (%) | 331 (27.6) |  |
| 2 n (%) | 483 (40.3) |  |
| 3 or more n (%) | 204 (17) |  |
| **Number of cesarian section** |  | 1194 |
| 0 n (%) | 856 (71.7) |  |
| 1 n (%) | 213(17.9) |  |
| 2 n(%) | 103 (8.6) |  |
| 3 or more n (%) | 22 (1.8) |  |
| **History of episiotomy n (%)** | 627 (62.3) | 1006 |
| **Perineal laceration during delivery n(%)** | 449 (37.4) | 1202 |
| **Hysterectomy n (%)** | 102 (5.66) | 1802 |
| **Organ prolapse surgery n (%)** | 17 (0.9) | 1816 |

**Table S5** Gynecological history of female participants. Number of vaginal delivery and cesarian section correspond to the number of children born by this route of delivery. Right column n: number of complete case analyzed per variable.
