# Supplementary material for: Anorectal incontinence among a working‐age population: A cross‐sectional survey of prevalence and epidemiology
Source: Colorectal Dis. 2026 Feb 5;28(2):e70392. doi: 10.1111/codi.70392 (PMC12876054; doi:10.1111/codi.70392)
Supplement: Supplementary file 8 — Table S6. [file CODI-28-0-s002.docx]

|  |  | | p value | n |
| --- | --- | --- | --- | --- |
|  | **Women** | **Men** |  |  |
| **Jorge-Wexner score, mean (SD)** | 1.5 (0.5) | 1.1 (0.7) | <0.001 | 2525 |
| Jorge-Wexner item 1 (gas leak), mean (SD) | 1.1 (1.2) | 0.78 (1.1) | <0.001 | 2532 |
| Jorge-Wexner item 2 (liquid stool leak), mean (SD) | 0.2 (0.5) | 0.2 (0.5) | 0.798 | 2531 |
| Jorge-Wexner item 3 (solid stool leak), mean (SD) | 0.6 (0.3) | 0.4 (0.3) | 0.190 | 2529 |
| Jorge-Wexner item 4 (wearing pad), mean (SD) | 0.04 (0.3) | 0.02 (0.3) | 0.103 | 2531 |
| Jorge-Wexner item 5 (quality of life impairment), mean (SD) | 0.2 (0.6) | 0.1 (0.5) | 0.006 | 2530 |
| **Anal incontinence, even rarely % [95% CI]** | 58.2 [56-60.5] | 44.7 [41.1-48.3] | <0.001 | 2528 |
| **Anal incontinence, even occasionally % [95% CI]** | 32.2 [30-34.4] | 21 [18-23.9] | <0.001 | 2528 |
| **Fecal incontinence, even rarely % [95% CI]** | 15.4 [13.8-17.1] | 13.8 [11.3-16.4] | 0.318 | 2528 |
| **Fecal incontinence, even occasionally % [95% CI]** | 6 [4.9-7.1] | 5 [3.4-6.6] | 0.336 | 2528 |
| **Fecal urgency** |  |  |  |  |
| Never n (%) | 56 | 59.9 | 0.025 | 2529 |
| < 1x/week n (%) | 36.7 | 35.5 |  |  |
| 1x/week or more n (%) | 7.3 | 4.6 |  |  |
| **Soiling % [95% CI]** | 6.5 [5.4-7.7] | 10.3 [8-12.5] | 0.001 | 2520 |
| **Reporting fecal incontinence according to Rome IV [95% CI]** | 4.4 [3.4-5.3] | 3.5 [2.2-4.9] | 0.331 | 2521 |
| **Rome IV fecal incontinence “criteria for research” % [95% CI]** | 2.5 [1.8-3.2] | 2 [0.9-3] | 0.436 | 2521 |

**Table S6**

Sensitivity analysis investigating the effect of gender on items of the questionnaire. Student T test was used to compare mean of Jorge-Wexner score items between men and women. Chi2 or T test when appropriate test was used for the other variables. Right column n: number of complete case analyzed per variable.
