# Supplementary material for: Anorectal incontinence among a working‐age population: A cross‐sectional survey of prevalence and epidemiology
Source: Colorectal Dis. 2026 Feb 5;28(2):e70392. doi: 10.1111/codi.70392 (PMC12876054; doi:10.1111/codi.70392)
Supplement: Supplementary file 9 — Table S7. [file CODI-28-0-s011.docx]

| n | 2525 | |  | 2528 | |  | 2528 | |  |
| --- | --- | --- | --- | --- | --- | --- | --- | --- | --- |
|  | **Women** | **Men** |  | **Women** | **Men** |  | **Women** | **Men** |  |
| Category of age | **Jorge-Wexner score, mean (SD**) | | **p** | **Anal incontinence, even rarely % [95% CI]** | | **p** | **Anal incontinence, even occasionally % [95% CI]** | | **p** |
| <25 yo | 1 (1.4) | 1.5 (2.3) | 0.272 | 44.4 [29.9-59] | 44.4 [29.9-59] | 0.437 | 28.9 [15.6-42.1] | 27.3 [0.9-4.6] | 0.890 |
|  | 1.2 (1.7) | |  | 47.8 [35.8-59.7] | |  | 28.4 [17.6-] | |  |
| 25-34 yo | 1.1 (1.7) | 0.7 (1.2) | **0.024** | 49.6 [44.8-54.5] | 39.8 [32.2-47.3] | **0.033** | 23.1 [19-27.2] | 16.2 [10.5-21.9] | 0.068 |
|  | 1 (1.6) | |  | 46.8 [42.7-50.9] | |  | 21.1 [17.8-24.8] | |  |
| 35-44 yo | 1.3 (1.8) | 0.8 (1.2) | **<0.001** | 57.8 [53-62.4] | 40.8 [33.5-48.1] | **<0.001** | 27.2 [23-31.5] | 13.2 [8.2-18.3] | **<0.001** |
|  | 1.1 (1.6) | |  | 52.8 [48.8-56.9] | |  | 23.2 [19.8-26.5] | |  |
| 45-49 yo | 1.9 (2.3) | 1 (1.6) | **<0.001** | 67.9 [62.1-73.7] | 40.2 [30.5-50] | **<0.001** | 40.2 [34.1-46.4] | 16.5 [9.1-23.9] | **<0.001** |
|  | 1.6 (2.2) | |  | 60.1 [54.8-65.2] | |  | 33.5 [28.5-38.5] | |  |
| 50-54 yo | 1.7 (2.1) | 1.3 (2.5) | 0.172 | 61.2 [55.3-67.1] | 48.5 [38.8-58.3] | **0.028** | 35.7 [30-41.5] | 24.8 [16.3-33.2] | **0.045** |
|  | 1.6 (2.2) | |  | 57.7 [52.6-62.7] | |  | 32.7 [27.9-37.5] | |  |
| 55-59 yo | 1.9 (2.4) | 1.7 (2.7) | 0.349 | 61.6 [56.1—67.1] | 50 [40.9-59.1] | **0.031** | 40.4 [34.9-45.9] | 31 [22.6-39.5] | 0.077 |
|  | 1.9 (2.5) | |  | 58.4 [53.7-63.1] | |  | 37.8 [33.2-42.5] | |  |
| >60 yo | 1.7 (2.1) | 2.1 (2.8) | 0.218 | 59 [50.3-67.7] | 60 [45.7-74.3] | 0.909 | 36.9 [28.3-45.5] | 73 [25.7-54.3] | 0.713 |
|  | 1.8 (2.3) | |  | 59.3 [51.8-66.7] | |  | 37.7 [30.4-45.1] | |  |
| p | <0.001 | <0.001 |  | <0.001 | 0.105 |  | <0.001 | <0.001 |  |
|  | <0.001 | |  | <0.001 | |  | <0.001 | |  |

| n | 2528 | |  | 2528 | |  | 2521 | |  |
| --- | --- | --- | --- | --- | --- | --- | --- | --- | --- |
|  | **Women** | **Men** |  | **Women** | **Men** |  | **Women** | **Men** |  |
| Category of age | **Fecal incontinence, even rarely % [95% CI]** | | **p** | **Fecal incontinence, even occasionally % [95% CI]** | | **p** | **Reporting fecal incontinence according to Rome % [95% CI]** | | **p** |
| <25 yo | 4.4 [0-10.5] | 9.1 [0-21.1] | 0.593 | 22.2 [0-6.5] | 4.5 [0-13.3] | 1 | 0 | 4.5 [0-13.3] | 0.338 |
|  | 6 [0.3-11.6 ] | |  | 3 [0-7.1] | |  | 1.6 [0-4.5] | |  |
| 25-34 yo | 9.8 [6.9-12.7] | 6.8 [29.4-10.7] | 0.260 | 3.7 [1.9-5.5] | 1.25 [0-3] | 0.172 | 3.4 [1.7-5.2] | 1.9 [0-4] | 0.420 |
|  | 9 [6.6-11.3] | |  | 3 [1.6-4.4] | |  | 3 [1.6-4.4] | |  |
| 35-44 yo | 14.3 [10.1-17.7] | 9.2 [4.9-13.5] | 0.089 | 3.8 [2-5.6] | 3.5 [0.7-6.2] | 0.856 | 3.3 [1.6-5] | 1.7 [0-3.7] | 0.419 |
|  | 12.8 [10.2-15.5] | |  | 3.7 [2.2-5.2] | |  | 2.8 [1.5-4.2] | |  |
| 45-49 yo | 20.3 [15.3-25.4] | 19.6 [11.7-27.5] | 0.878 | 6.5 [3.4-9.6] | 5.2 [0.8-9.6] | 0.639 | 5.3 [2.5-8.1] | 4.1 [0.2-8.2] | 0.788 |
|  | 20.1 [15.9-24.4] | |  | 6.1 [3.6-8.7] | |  | 5 [2.7-7.3] | |  |
| 50-54 yo | 15.2 [10.9-19.6] | 18.8 [11.2-26.4] | 0.404 | 8.4 [5-1.2] | 6.9 [2-11.9] | 0.651 | 5.7 [2.9-8.5] | 4 [0.2-7.9] | 0.527 |
|  | 16.2 [12.4-19.9] | |  | 8 [5.2-10.8] | |  | 5.3 [3-7.6] | |  |
| 55-59 yo | 19.5 [15.1-24] | 17.2 [10.4-24.1] | 0.592 | 8.9 [5.7-12.2] | 7.8 [2.9-12.6] | 0.700 | 6.3 [3.5-9] | 6 [1.7-10.4] | 0.929 |
|  | 18.9 [15.1-22.7] | |  | 8.6 [5.9-11.3] | |  | 6.2 [3.9-8.5] | |  |
| >60 yo | 22.1 [14.8-29.5] | 26.7 [13.8-39.6] | 0.539 | 9.8 [4.5-15.1] | 13.3 [3.4-23.3] | 0.576 | 3.3 [0.1-6.5] | 6.7 [0-14] | 0.392 |
|  | 23.4 [16.9-29.8] | |  | 10.8 [6.1-15.5] | |  | 4.2 [1.2-7.3] | |  |
| p | **<0.001** | NA* |  | NA* | **0.014** |  | 0.224 | 0.228 |  |
|  | **<0.001** | |  | NA* | |  | 0.069 | |  |

| n | 2521 | |  | 2520 | |  |
| --- | --- | --- | --- | --- | --- | --- |
|  | **Women** | **Men** |  | **Women** | **Men** |  |
| Category of age | **Rome IV fecal incontinence % [95% CI]** | | **p** | **Soiling % [95% CI]** | | **p** |
| <25 yo | 0 | 4.6 [0-13.3] | 0.338 | 2.3 [0-6.8] | 13.6 [0-28] | 0.109 |
|  | 1.5 [0-4.5] | |  | 6.2 [3.1-12] | |  |
| 25-34 yo | 2.2 [0.8-3.6] | 0.6 [0-1.8] | 0.296 | 5.2 [3-7.3] | 9.3 [4.8-13.8] | 0.068 |
|  | 1.8 [0.7-2.8] | |  | 6.4 [4.3-8.4] | |  |
| 35-44 yo | 0.9 [0-1.9] | 0 | 0.583 | 5.9 [3.6-8.1] | 8.7 [4.5-12.9] | 0.210 |
|  | 0.7 [0.2-1.3] | |  | 6.7 [4.7-8.7] | |  |
| 45-49 yo | 2.5 [0.5-4.4] | 2 [0-4.9] | 1 | 9 [5.4-12.6] | 10.4 [4.3-16.5] | 0.682 |
|  | 2.3 [0.7-4] | |  | 9.4 [6.3-12.5] | |  |
| 50-54 yo | 3.8 [1.5-6.1] | 3 [0-6.4] | 1 | 4.9 [2.3-7.6] | 7.1 [2-12.1] | 0.430 |
|  | 4 [1.7-5.5] | |  | 5.5 [3.2-7.9] | |  |
| 55-59 yo | 4.6 [2.3-7] | 4.3 [0.6-8] | 0.891 | 8.6 [5.4-11.7] | 12.9 [6.8-19] | 0.180 |
|  | 4.5 [2.5-6.5] | |  | 9.8 [6.9-12.6] | |  |
| >60 yo | 1.7 [0-4] | 4.4 [0-10.4] | 0.300 | 8.3 [3.4-13.3] | 5.7 [6.6-29] | 0.096 |
|  | 2.4 [0.8-4.8] | |  | 10.9 [6.2-15.7] | |  |
| p | **0.043** | **0.017** |  | 0.190 | 0.435 |  |
|  | **0.002** | |  | NA | |  |

**Table S7**

Primary and sensitivity analysis: Relation between age category and Wexner score, rare or occasional anal or fecal incontinence, Rome IV criteria and soiling. P value was calculated with t test for mean comparison or Chi2/Fisher tests for proportion comparison between gender at different age category. Mean was compared in different age category using ANOVA test. Proportions were compared in different age category Chi2/Fisher tests. NA : not applicable *Fisher test required but calculation not possible.
