# Supplementary material for: Anorectal incontinence among a working‐age population: A cross‐sectional survey of prevalence and epidemiology
Source: Colorectal Dis. 2026 Feb 5;28(2):e70392. doi: 10.1111/codi.70392 (PMC12876054; doi:10.1111/codi.70392)
Supplement: Supplementary file 10 — Table S8. [file CODI-28-0-s013.docx]

| Diabetes | | | | | | |
| --- | --- | --- | --- | --- | --- | --- |
|  | Univariate logistic regression | | | Multivariate logistic regression | | |
|  | OR | 95% CI | p value | OR | 95% CI | P value |
| Soiling | 2.29 | 1.01-5.23 | **0.047** | 1.43 | 0.59-3.45 | 0.431 |
| Rome IV fecal incontinence criteria for research | 4.3 | 1.49-12.4 | **0.007** | 3.3 | 1.09-10.08 | **0.035** |
| Jorge-Wexner ≥ 3 | 1.2 | 0.62-2.46 | 0.544 | 0.9 | 0.45-1.89 | 0.828 |

**Table S8** Association between incontinence items and the presence of diabetes. Adjustment for sex, age and BMI n= 2498 Highlighted in green are rows where association was confirmed with multivariate analysis.
