# Supplementary material for: Anorectal incontinence among a working‐age population: A cross‐sectional survey of prevalence and epidemiology
Source: Colorectal Dis. 2026 Feb 5;28(2):e70392. doi: 10.1111/codi.70392 (PMC12876054; doi:10.1111/codi.70392)
Supplement: Supplementary file 11 — Table S9. [file CODI-28-0-s001.docx]

|  |  | Univariate logistic regression | | |  |
| --- | --- | --- | --- | --- | --- |
|  | Items | OR | 95% CI | p value | n |
| **BMI <18.5** | Soiling | 0.21 | 0.03-1.56 | 0.126 | 2501 |
|  | Reporting fecal incontinence according to Rome | 0.33 | 0.05-2.44 | 0.279 | 2501 |
|  | Rome IV fecal incontinence | 0.62 | 0.83-4.6 | 0.641 | 2480 |
|  | Jorge-Wexner ≥ 3 | 0.52 | 0.25-1.1 | 0.089 | 2496 |
| **BMI 18.5-25** | Soiling | 1 | NA | 1 | 2501 |
|  | Reporting fecal incontinence according to Rome | 1 | NA | 1 | 2501 |
|  | Rome IV fecal incontinence | 1 | NA | 1 | 2480 |
|  | Jorge-Wexner ≥ 3 | 1 | NA | 1 | 2496 |
| **BMI 25-30** | Soiling | 1.2 | 0.84-1.7 | 0.318 | 2501 |
|  | Reporting fecal incontinence according to Rome | 0.9 | 0.57-1.43 | 0.655 | 2501 |
|  | Rome IV fecal incontinence | 0.89 | 0.48-1.66 | 0.708 | 2480 |
|  | Jorge-Wexner ≥ 3 | 0.52 | 0.25-1.10 | 0.089 | 2496 |
| **BMI 30-35** | Soiling | 2.02 | 1.27-3.22 | **0.03** | 2501 |
|  | Reporting fecal incontinence according to Rome | 0.81 | 0.71-5.82 | 0.188 | 2501 |
|  | Rome IV fecal incontinence | 1.28 | 0.53-3.08 | 0.581 | 2480 |
|  | Jorge-Wexner ≥ 3 | 1.3 | 0.92-1.83 | 0.139 | 2496 |
| **BMI 35-40** | Soiling | 2.85 | 1.3-6.25 | **0.009** | 2501 |
|  | Reporting fecal incontinence according to Rome | 2.03 | 0.71-5.82 | 0.188 | 2501 |
|  | Rome IV fecal incontinence | 1.81 | 0.43-7.75 | 0.424 | 2480 |
|  | Jorge-Wexner ≥ 3 | 2.15 | 1.17-3.9 | **0.013** | 2496 |
| **BMI>40** | Soiling | 4.45 | 1.6-12.39 | **0.004** | 2501 |
|  | Reporting fecal incontinence according to Rome | 1.12 | 0.15-8.44 | 0.916 | 2501 |
|  | Rome IV fecal incontinence | 1 | NA | NA | 2480 |
|  | Jorge-Wexner ≥ 3 | 1.22 | 0.45-3.37 | 0.696 | 2496 |

***Table S9***

*Effect of body mass index (BMI) on anal incontinence prevalence items. A: Anal incontinence, even rarely ,B: Anal incontinence, even occasionally, C:, D:, E:, F:, G: H:, NA: not applicable. *model not valid. P value under 0.05 are in bold.*
