# Supplementary material for: Anorectal incontinence among a working‐age population: A cross‐sectional survey of prevalence and epidemiology
Source: Colorectal Dis. 2026 Feb 5;28(2):e70392. doi: 10.1111/codi.70392 (PMC12876054; doi:10.1111/codi.70392)
Supplement: Supplementary file 12 — Table S10. [file CODI-28-0-s007.docx]

|  |  | Univariate logistic regression | | | Multivariate logistic regression | | | n |
| --- | --- | --- | --- | --- | --- | --- | --- | --- |
| Vaginal delivery |  | OR | 95% CI | p value | OR | 95% CI | p value |  |
| Anal incontinence, even rarely | Primipartum | 1.11 | 0.85-1.46 | 0.445 | 0.93 | 0.7-1.24 | 0.629 | 1616 |
|  | Multipartum | 1.49 | 1.19-1.86 | **0.001** | 1.16 | 0.89-1.5 | 0.281 |  |
| Anal incontinence, even occasionally | Primipartum | 1.31 | 0.97-1.76 | 0.076 | 1.14 | 0.83-1.57 | 0.408 | 1615 |
|  | Multipartum | 1.79 | 1.41-2.28 | **<0.001** | 1.46 | 1.1-1.92 | 0.008 |  |
| Fecal incontinence, even rarely | Primipartum | 1.4 | 0.95-2.06 | 0.085 | 1.09 | 0.73-1.63 | 0.682* | 1.615 |
|  | Multipartum | 1.68 | 1.23-2.29 | 0.001 | 1.2 | 0.84-1.71 | 0.307* |  |
| Fecal incontinence, even occasionally | Primipartum | 1.97 | 1.08-3.58 | **0.028** | 1.64 | 0.86-3.1 | 0.131 | 1615 |
|  | Multipartum | 2.19 | 1.32-3.65 | **0.003** | 1.69 | 0.95-3.02 | 0.075 |  |
| Soiling | Primipartum | 1.37 | 0.8-2.33 | 0.255 | 1.13 | 0.64-1.99 | 0.665 | 1616 |
|  | Multipartum | 1.31 | 0.83-2.05 | 0.244 | 1.02 | 0.61-1.69 | 0.951 |  |
| Reporting fecal incontinence according to Rome | Primipartum | 1.75 | 0.87-3.5 | 0.116 | 1.43 | 0.69-2.96 | 0.340* | 1573 |
|  | Multipartum | 2.06 | 1.2-3.69 | **0.014** | 1.57 | 0.82-3.1 | 0.173* |  |
| Rome IV fecal incontinence | Primipartum | 2.26 | 0.93-5.52 | 0.072 | 2.05 | 0.8-5.27 | 0.135* | 1573 |
|  | Multipartum | 2.17 | 0.99-4.76 | 0.052 | 1.88 | 0.77-4.58 | 0.166* |  |
| Wexner ≥ 3 | Primipartum | 1.6 | 1.15-2.22 | 0.006 | 1.26 | 0.89-1.8 | 0.198 | 1613 |
|  | Multipartum | 1.87 | 1.43-2.46 | **<0.001** | 1.32 | 0.96-1.8 | 0.084 |  |

**Table S10**

Primary and sensitivity analysis. Compared to nulliparous women, mothers of multiple birth by vaginal delivery had a slight increased association wit anal or fecal incontinence. However, this association disappeared after adjustment for age category *Model not valid.
