# Supplementary material for: Anorectal incontinence among a working‐age population: A cross‐sectional survey of prevalence and epidemiology
Source: Colorectal Dis. 2026 Feb 5;28(2):e70392. doi: 10.1111/codi.70392 (PMC12876054; doi:10.1111/codi.70392)
Supplement: Supplementary file 13 — Table S11. [file CODI-28-0-s003.docx]

|  |  | **Univariate logistic regression** | | | **Multivariate logistic regression** | | | n |
| --- | --- | --- | --- | --- | --- | --- | --- | --- |
|  | **Number of episiotomy** | **OR** | **95% CI** | **p value** | **OR** | **95% CI** | **p value** |  |
| **Anal incontinence, even rarely** | 1 | 1 | 0.75-1.33 | 0.991 | 0.98 | 0.73-1.32 | 0.898 | 999 |
|  | 2 | 1.01 | 0.73-1.53 | 0.777 | 1.03 | 0.69-1.53 | 0.887 |  |
|  | ≥3 | 1.25 | 0.6-2.58 | 0.552 | 1.22 | 0.58-2.57 | 0.604 |  |
| **Anal incontinence, even occasionally** | 1 | 1 | 0.75-1.34 | 0.980 | 0.91 | 0.67-1.23 | 0.534 | 999 |
|  | 2 | 1.01 | 0.73-1.54 | 0.772 | 0.88 | 0.59-1.32 | 0.544 |  |
|  | ≥3 | 1.7 | 0.85-3.41 | 0.135 | 1.59 | 0.78-3.25 | 0.206 |  |
| **Fecal incontinence, even rarely** | 1 | 0.85 | 0.59-1.23 | 0.389 | 0.78 | 0.54-1.42 | 0.203 | 999 |
|  | 2 | 0.93 | 0.58-1.48 | 0.753 | 0.83 | 0.51-1.36 | 0.460 |  |
|  | ≥3 | 1.26 | 0.55-2.89 | 0.586 | 1.16 | 0.5-2.71 | 0.734 |  |
| **Fecal incontinence, even occasionally** | 1 | 0.8 | 0.47-1.36 | 0.414 | 0.71 | 0.41-1.23 | 0.220 | 993 |
|  | 2 | 1.06 | 0.56-2.02 | 0.853 | 0.88 | 0.44-1.75 | 0.714 |  |
|  | ≥3 | 1.05 | 0.3-3.62 | 0.939 | 0.89 | 0.25-3.15 | 0.853 |  |
| **Soiling** | 1 | 0.9 | 0.53-1.54 | 0.699 | 0.83 | 0.48-1.46 | 0.519 | 999 |
|  | 2 | 1.15 | 0.6-2.2 | 0.682 | 1.07 | 0.54-2.15 | 0.840 |  |
|  | ≥3 | 0.73 | 0.17-3.03 | 0.678 | 0.73 | 0.16-3.31 | 0.684 |  |
| **Reporting fecal incontinence according to Rome** | 1 | 0.86 | 0.47-1.57 | 0.623 | 0.83 | 0.45-1.6 | 0.565 | 993 |
|  | 2 | 0.99 | 0.44-2.04 | 0.895 | 0.94 | 0.42-2.12 | 0.878 |  |
|  | ≥3 | 0.46 | 0.06-3.48 | 0.448 | 0.42 | 0.05-3.27 | 0.405 |  |
| **Rome IV fecal incontinence** | 1 | 1.37 | 0.61-3.1 | 0.444 | 1.37 | 0.58-3.22 | 0.470 | 993 |
|  | 2 | 1.56 | 0.58-4.2 | 0.378 | 1.6 | 0.55-4.65 | 0.384 |  |
|  | ≥3 | 1.09 | 0.14-8.73 | 0.939 | 0.98 | 0.11-8.5 | 0.986 |  |
| **Jorge- Wexner ≥ 3** | 1 | 1.02 | 0.74-1.39 | 0.914 | 0.89 | 0.64-1.23 | 0.473 | 997 |
|  | 2 | 0.93 | 0.61-1.4 | 0.718 | 0.72 | 0.46-1.13 | 0.151 |  |
|  | ≥3 | 1.45 | 0.7-3.02 | 0.321 | 1.28 | 0.6-2.73 | 0.530 |  |

**Table S11** Primary and sensitivity analysis. Association between number of episiotomy and the presence of fecal/anal incontinence panel. Adjustment for age category, BMI category and presence of a diabetes
