# Supplementary material for: Anorectal incontinence among a working‐age population: A cross‐sectional survey of prevalence and epidemiology
Source: Colorectal Dis. 2026 Feb 5;28(2):e70392. doi: 10.1111/codi.70392 (PMC12876054; doi:10.1111/codi.70392)
Supplement: Supplementary file 14 — Table S12. [file CODI-28-0-s004.docx]

|  | Univariate logistic regression | | | Multivariate logistic regression | | | n |
| --- | --- | --- | --- | --- | --- | --- | --- |
| Night shift | OR | 95% CI | p value | OR | 95% CI | P value |  |
| Anal incontinence, even rarely | 0.9 | 0.76-1.06 | 0.201 | 1 | 0.84-1.18 | 0.980 | 2515 |
| Anal incontinence, even occasionally | 0.71 | 0.59-0.85 | **<0.001** | 0.84 | 0.7-1.101 | 0.065 | 2515 |
| Fecal incontinence, even rarely | 0.74 | 0.59-0.94 | **0.013** | 0.88 | 0.69-1.26 | 0.311 | 2515 |
| Fecal incontinence, even occasionally | 0.55 | 0.38-0.81 | **0.003** | 0.69 | 0.47-1.04 | 0.074 | 2515 |
| Soiling | 0.79 | 0.58-1.09 | 0.149 | 0.85 | 0.61-1.19 | 0.346 | 2517 |
| Reporting fecal incontinence according to Rome | 0.83 | 0.55-1.26 | 0.377 | 0.95 | 0.62-1.48 | 0.832 | 2516 |
| Rome IV fecal incontinence | 0.75 | 0.43 | 0.31-1.31 | 0.93 | 0.52-1.68 | 0.813 | 2516 |
| Jorge-Wexner ≥ 3 | 0.63 | 0.51-0.78 | **<0.001** | 0.79 | 0.64-0.99 | **0.038** | 2512 |

**Table S12** Primary and sensitivity analysis. Association between night shift and anal incontinence panel items. Adjustment for age. *model not valid. Bold highlight p value under 0.005.
