# Supplementary material for: Anorectal incontinence among a working‐age population: A cross‐sectional survey of prevalence and epidemiology
Source: Colorectal Dis. 2026 Feb 5;28(2):e70392. doi: 10.1111/codi.70392 (PMC12876054; doi:10.1111/codi.70392)
Supplement: Supplementary file 15 — Table S13. [file CODI-28-0-s006.docx]

|  |  | Mulivariate logistic regression | | | n |
| --- | --- | --- | --- | --- | --- |
|  | Items | OR | 95% CI | p value |  |
| **Nursing staff** | Soiling | 1 | NA | 1 | 2500 |
|  | Reporting fecal incontinence according to Rome | 1 | NA | 1 | 2501 |
|  | Rome IV fecal incontinence | 1 | NA | 1 | 2452 |
|  | Jorge-Wexner ≥ 3 | 1 | NA | 1 | 2525 |
| **Administration staff** | Soiling | 1.2 | 0.78-1.85 | 0.413 | 2500 |
|  | Reporting fecal incontinence according to Rome | 1.11 | 0.63-1.99 | 0.714 | 2501 |
|  | Rome IV fecal incontinence | 1.55 | 0.75-3.18 | 0.233 | 2452 |
|  | Jorge-Wexner ≥ 3 | 1.47 | 1.11-1.93 | 0.007* | 2525 |
| **Medical staff** | Soiling | 0.98 | 0.61-1.58 | 0.937 | 2500 |
|  | Reporting fecal incontinence according to Rome | 0.88 | 0.44-1.75 | 0.715 | 2501 |
|  | Rome IV fecal incontinence | 0.64 | 0.22-1.90 | 0.423 | 2452 |
|  | Jorge-Wexner ≥ 3 | 0.82 | 0.58-1.14 | 0.233* | 2525 |
| **Medical technical staff** | Soiling | 0.69 | 0.36-1.36 | 0.281 | 2500 |
|  | Reporting fecal incontinence according to Rome | 0.81 | 0.34-1.9 | 0.629 | 2501 |
|  | Rome IV fecal incontinence | 0.8 | 0.24-2.72 | 0.722 | 2452 |
|  | Jorge-Wexner ≥ 3 | 1.2 | 0.82-1.75 | 0.349* | 2525 |
| **Therapy staff** | Soiling | 1.43 | 0.81-2.52 | 0.221 | 2500 |
|  | Reporting fecal incontinence according to Rome | 1.73 | 0.89-3.37 | 0.105 | 2501 |
|  | Rome IV fecal incontinence | 1.65 | 0.24-2.72 | 0.722 | 2452 |
|  | Jorge-Wexner ≥ 3 | 1.27 | 0.87-1.86 | 0.219* | 2525 |

|  |  | Multivariate logistic regression | | | n |
| --- | --- | --- | --- | --- | --- |
|  | Items | OR | 95% CI | p value |  |
| **Technical staff** | Soiling | 1.18 | 0.61-2.29 | 0.620 | 2500 |
|  | Reporting fecal incontinence according to Rome | 2.15 | 0.91-5.07 | 0.082 | 2501 |
|  | Rome IV fecal incontinence | 3.42 | 1.2-9.76 | **0.022** | 2452 |
|  | Jorge-Wexner ≥ 3 | 1.66 | 1.02-20.71 | 0.041* | 2525 |
| **Logistic**  **staff** | Soiling | 1.31 | 0.5-3.45 | 0.580 | 2500 |
|  | Reporting fecal incontinence according to Rome | 0.56 | 0.07-4.16 | 0.569 | 2501 |
|  | Rome IV fecal incontinence | 1 | NA | NA | 2452 |
|  | Jorge-Wexner ≥ 3 | 2.36 | 1.26-4.42 | 0.007* | 2525 |
| **Cleaning staff and kitchen staff** | Soiling | 1.82 | 0.74-4.48 | 0.193 | 2500 |
|  | Reporting fecal incontinence according to Rome | 1.73 | 0.89-3.37 | 0.105 | 2501 |
|  | Rome IV fecal incontinence | 3.87 | 1.01-13.67 | **0.035** | 2452 |
|  | Jorge-Wexner ≥ 3 | 3.51 | 1.89-6.52 | <0.001* | 2525 |
| **Social staff** | Soiling | 1 | NA | NA | 2500 |
|  | Reporting fecal incontinence according to Rome | 1 | NA | NA | 2501 |
|  | Rome IV fecal incontinence | 1 | NA | NA | 2452 |
|  | Jorge-Wexner ≥ 3 | 0.21 | 1.89-6.52 | <0.001* | 2525 |
| **Other** | Soiling | 1.21 | 0.59-2.13 | 0.726 | 2500 |
|  | Reporting fecal incontinence according to Rome | 0.86 | 0.34-2.28 | 0.764 | 2501 |
|  | Rome IV fecal incontinence | 1.38 | 0.47-4.1 | 0.558 | 2452 |
|  | Jorge-Wexner ≥ 3 | 1.13 | 0.74-1.73 | 0.560* | 2525 |

**Table S13**

Occupational effect on anal incontinence prevalence. Adjustment for gender. NA: not applicable. *model not valid. P value under 0.05 are in bold.
