# Supplementary material for: Anorectal incontinence among a working‐age population: A cross‐sectional survey of prevalence and epidemiology
Source: Colorectal Dis. 2026 Feb 5;28(2):e70392. doi: 10.1111/codi.70392 (PMC12876054; doi:10.1111/codi.70392)
Supplement: Supplementary file 16 — Table S14. [file CODI-28-0-s005.docx]

| Cesarean section exclusively | Univariate logistic regression | | | Multivariate logistic regression | | |  |
| --- | --- | --- | --- | --- | --- | --- | --- |
|  | **OR** | **95% CI** | **p value** | **OR** | **95% CI** | **p value** | **n** |
| Anal incontinence, even rarely | 0.98 | 0.72-1.34 | 0.898 | 0.96 | 0.7-1.31 | 0.788* | 1811 |
| Anal incontinence, even occasionally | 0.87 | 0.62-1.21 | 0.400 | 0.84 | 0.6-1.17 | 0.302 | 1811 |
| Fecal incontinence, even rarely | 0.83 | 0.53-1.3 | 0.419 | 0.8 | 0.51-1.26 | 0.339* | 1811 |
| Fecal incontinence, even occasionally | 0.81 | 0.4-1.63 | 0.547 | 0.77 | 0.38-1.56 | 0.473 | 1811 |
| Soiling | 0.64 | 0.31-1.34 | 0.237 | 0.63 | 0.3-1.31 | 0.214 | 1807 |
| Reporting fecal incontinence according to Rome | 0.74 | 0.32-1.71 | 0.476 | 0.72 | 0.31-1.67 | 0.438* | 1808 |
| Rome IV fecal incontinence | 0.64 | 0.2-2.09 | 0.460 | 0.62 | 0.19-2.02 | 0.425 | 1808 |
| Wexner ≥ 3 | 0.85 | 0.58-1.24 | 0.390 | 0.82 | 0.55-1.2 | 0.291* | 1809 |

**Table S14**. Primary and sensitivity analysis. Effect of cesarean section on anorectal incontinence. Adjustement for age category. *model not valid
