# Supplementary material for: Anorectal incontinence among a working‐age population: A cross‐sectional survey of prevalence and epidemiology
Source: Colorectal Dis. 2026 Feb 5;28(2):e70392. doi: 10.1111/codi.70392 (PMC12876054; doi:10.1111/codi.70392)
Supplement: Supplementary file 17 — Data S1. [file CODI-28-0-s009.pdf]

Prévalence de l'incontinence anale

Veuillez remplir le questionnaire ci-dessous.

Chères Collègues, Chers Collègues,

Nous vous remercions d'avoir accepté de participer à cette enquête en ligne. Pour rappel, les réponses données ci-dessous sont totalement anonymes puisqu'il n'y a aucune moyen de vous identifier, ni par les informations données, ni de façon informatique, l'adresse de votre ordinateur (adresse IP) n'étant pas enregistrée.

Le but de cette enquête est de déterminer la fréquence de l'incontinence anale et ses symptômes chez des travailleuses et travailleurs d'une structure hospitalière grâce à des questions explorant différents aspects de l'incontinence anale. A la fin du questionnaire, des questions sur vos antécédents ainsi que sur le corps de métier auquel vous appartenez vous seront posées.

Nous vous remercions d'avance de ne répondre au questionnaire qu'une seule fois et de façon sincère.

Êtes-vous biologiquement:

- ◯ Une femme
- ◯ Un homme

A quelle catégorie d'âge appartenez-vous?

- ◯ Moins de 25 ans
- ◯ Entre 25 et 34 ans
- ◯ Entre 35 et 44 ans
- ◯ Entre 45 et 49 ans
- ◯ Entre 50 et 54 ans
- ◯ Entre 55 et 59 ans
- ◯ Plus de 60 ans

Les questions suivantes traitent des symptômes éventuels d'incontinence que vous pourriez avoir; répondez en toute sincérité.

Vous arrive-t-il d'avoir des fuites anales incontrôlées de flatulences (gaz intestinaux) ?

- ◯ Jamais
- ◯ Rarement : moins de une fois par mois (< 1x/mois)
- ◯ Parfois : une fois par mois ou plus, mais moins de une fois par semaine (≥1x/mois-< 1x/semaine)
- ◯ Souvent : une fois par semaine ou plus, mais moins de une fois par jour (≥1x/semaine-< 1x/jour)
- ◯ Toujours : une fois par jour ou plus (≥1x/jour)

Vous arrive-t-il d'avoir des fuites anales incontrôlées de selles liquides ?

- ◯ Jamais
- ◯ Rarement : moins de une fois par mois (< 1x/mois)
- ◯ Parfois : une fois par mois ou plus, mais moins de une fois par semaine (≥1x/mois-< 1x/semaine)
- ◯ Souvent : une fois par semaine ou plus, mais moins de une fois par jour (≥1x/semaine-< 1x/jour)
- ◯ Toujours : une fois par jour ou plus (≥1x/jour)

Vous arrive-t-il d'avoir des fuites anales incontrôlées de selles formées ?

- ◯ Jamais
- ◯ Rarement : moins de une fois par mois (< 1x/mois)
- ◯ Parfois : une fois par mois ou plus, mais moins de une fois par semaine (≥1x/mois-< 1x/semaine)
- ◯ Souvent : une fois par semaine ou plus, mais moins de une fois par jour (≥1x/semaine-< 1x/jour)
- ◯ Toujours : une fois par jour ou plus (≥1x/jour)

À cause de fuites anales, devez-vous porter des protections ?	◯ Jamais<br>◯ Rarement : moins de une fois par mois (< 1x/mois)<br>◯ Parfois : une fois par mois ou plus, mais moins de une fois par semaine (≥1x/mois-< 1x/semaine)<br>◯ Souvent : une fois par semaine ou plus, mais moins de une fois par jour (≥1x/semaine-< 1x/jour)<br>◯ Toujours : une fois par jour ou plus (≥1x/jour)
Les fuites anales ont-elles un retentissement sur la qualité de votre vie ?	◯ Jamais<br>◯ Rarement : moins de une fois par mois (< 1x/mois)<br>◯ Parfois : une fois par mois ou plus, mais moins de une fois par semaine (≥1x/mois-< 1x/semaine)<br>◯ Souvent : une fois par semaine ou plus, mais moins de une fois par jour (≥1x/semaine-< 1x/jour)<br>◯ Toujours : une fois par jour ou plus (≥1x/jour)
Vous arrive-t-il d'avoir une très forte envie au point de vous précipiter pour aller à la selle ?	◯ Non, jamais<br>◯ Oui, moins d'une fois par semaine (< 1x/semaine)<br>◯ Oui, au moins une fois par semaine (≥1x/semaine)

Les questions suivantes s'appliquent aux personnes ayant des symptômes d'incontinence. Si vous n'en avez pas, répondez simplement négativement aux questions.

Avez-vous des pertes anales incontrôlées de selles depuis plus de 6 mois ?	◯ Oui<br>◯ Non
Durant les derniers 3 mois combien avez-vous eu une fuite/pertes anales incontrôlées de selles ?	__________________________________
Avez-vous des suintements de l'anus (salissures) ?	◯ Oui<br>◯ Non
Avez-vous des pertes de selles qui passent inaperçues et que vous remarquez après coup (en dehors des suintements) ?	◯ Oui<br>◯ Non
À quelle fréquence allez-vous à la selle ?	◯ Plus de 7 fois par jour (24heures)<br>◯ 4 à 7 fois par jour (24heures)<br>◯ 1 à 3 fois par jour(24heures)<br>◯ Moins d'une fois par jour (24 heures)
Vous arrive-t-il de retourner à la selle moins d'une heure après y être allé ?	◯ Non, jamais<br>◯ Oui, moins d'une fois par semaine (< 1x/semaine)<br>◯ Oui, au moins une fois par semaine (≥1x/semaine)
Est-ce qu'il vous arrive d'utiliser des laxatifs et si oui à quelle fréquence ?	◯ Non, jamais<br>◯ Oui, moins d'une fois par semaine (< 1x/semaine)<br>◯ Oui, au moins une fois par semaine (≥1x/semaine)
Avez-vous eu des enfants?	◯ Oui<br>◯ Non
De combien d'enfant(s) avez-vous accouché par voie basse (voie naturelle) ?	__________________________________
De combien d'enfant(s) avez-vous accouché par césarienne ?	__________________________________

A-t-on dû pratiquer une épisiotomie lors de votre(s) accouchement(s) et combien de fois (indiquer le nombre d'épisiotomie que vous avez eue; si pas d'épisiotomie, indiquer 0) ?	__________________
Avez-vous bénéficié d'une hystérectomie (ablation de l'utérus)?	◯ Oui, par voie vaginale<br>◯ Oui, par voie abdominale<br>◯ Non
Avez-vous eu une déchirure du périnée lors de votre accouchement ?	◯ Oui<br>◯ Non
Avez-vous déjà eu des blessures ou traumatismes de la région autour de l'anus ou au niveau de l'anus par le passé, en dehors des déchirures de l'accouchement?	◯ Oui<br>◯ Non
Avez-vous déjà eu des blessures ou traumatismes de la région autour de l'anus ou au niveau de l'anus par le passé?	◯ Oui<br>◯ Non
Avez-vous déjà eu une chirurgie de l'anus/du périnée?	◯ Oui<br>◯ Non
Quel(-s) type(-s) de chirurgie? (plusieurs réponses possibles)	☐ Une chirurgie des hémorroïdes<br>☐ Oui, une chirurgie d'une fistule anale<br>☐ Oui, une chirurgie d'un abcès anal<br>☐ Oui, une chirurgie d'une fissure anale<br>☐ Oui, une chirurgie pour une descente d'organe
Souffrez-vous d'un diabète ?	◯ Oui<br>◯ Non
Depuis combien d'années souffrez-vous de diabète? (si moins de une année, indiquez 0)?	__________________
Quel est votre taille en cm ?	__________________
Quel est votre poids en kg ?	__________________
Fumez-vous ?	◯ Oui<br>◯ Non
Au cours des 12 deniers mois, avez-vous effectué un travail de nuit ?	◯ Oui<br>◯ Non

A quelle catégorie de collaborateur appartenez-vous (ne tient pas compte du niveau hiérarchique) ?

- ◯ Ambulancier, assistante en soins et santé communautaire
- ◯ Assistant social, animateur, aumônier, gardien de prison
- ◯ Auxiliaire de soin (aide en soin et accompagnement, aide-soignant, aide de salle)
- ◯ Buanderie, propreté et hygiène, stérilisation
- ◯ Chauffeur (transport de personne, marchandise, ambulance, hélicoptère)
- ◯ Diététicien, ergothérapeute, hygiéniste dentaire, pédicure
- ◯ Infirmière non-spécialiste
- ◯ Infirmière spécialiste et technicien en salle d'opération
- ◯ Infirmière de liaison/assistante de gestion/responsable flux patient, case manager, coordinatrice en transplantation, infirmière chargée de formation, IP manager, assistante clinique, responsable et adjoint de soins, responsable d'unité
- ◯ Informatique (analyste, informaticien, programmeur etc.)
- ◯ Médecin sans titre de spécialiste
- ◯ Médecin avec titre de spécialiste
- ◯ Personnel administratif (codage, commis administratif, comptable, direction, gestionnaire, secrétariat etc.)
- ◯ Personnel technique et maintenance (y compris : architecte/dessinateur en bâtiment, ingénieur, mécanicien, espaces verts)
- ◯ Personnel technique de laboratoire
- ◯ Personnel scientifique avec ou sans mandat de recherche : biologiste, biostatisticien, statisticien, physicien, chimiste, pharmacien
- ◯ Physiothérapeute
- ◯ Personnel de puériculture, éducateur, enseignant
- ◯ Psychologue, logopédiste, sociologue, thérapeute
- ◯ Sage femme
- ◯ Sécurité, logistique et intendance
- ◯ Technicien en radiologie médicale, assistant technique en EEG, assistant technique en audiologie, assistant technique en angiologie, cardio-technicien, technicien dentaire, technicien en fonction pulmonaire, orthoptiste, photographie médicale
- ◯ Restauration
- ◯ Réception, communication, huissier et téléopérateur
- ◯ Transporteur patient et marchandise (interne)
- ◯ Autre
